# Effectiveness of Apneic Oxygenation During Induction of General Anesthesia in Children Undergoing Adenotonsillectomy: A Prospective Randomized Controlled Study

**DOI:** 10.5812/aapm-169980

**Published:** 2026-02-27

**Authors:** Safaa Gaber Ragab, Ahmed Ali Lotfy, Joseph Makram Botros, Hasnaa Mohsen Hashem, Rana Ahmed Abdelghaffar, Doaa Lotfy Abdel Baky

**Affiliations:** 1 Faculty of Medicine, Fayoum University, Egypt

**Keywords:** Apneic Oxygenation, Pediatric Anesthesia, Adenotonsillar Hypertrophy, Oxygen Desaturation, Obstructive Airway, Tracheal Intubation

## Abstract

**Background:**

Children undergoing adenotonsillectomy often have partial airway obstruction due to hypertrophic tonsils and adenoids, increasing their risk of oxygen desaturation during anesthesia induction.

**Objectives:**

This study aimed to evaluate whether apneic oxygenation via nasal cannula prevents oxygen desaturation during tracheal intubation in children aged 3 - 10 years undergoing adenotonsillectomy while assessing its effects on intubation conditions and hemodynamic stability.

**Methods:**

In this prospective, single-blinded, randomized controlled trial, 140 children scheduled for adenotonsillectomy were allocated to either standard intubation (group A, n = 70) or apneic oxygenation (group B, n = 70; 0.2 L/kg/min via nasal cannula). The primary outcome was the lowest peripheral oxygen saturation (SpO₂) during intubation.

**Results:**

Peripheral SpO₂ was significantly lower during intubation in group A (97.40 ± 2.96) than in group B (99.91 ± 0.28) (P < 0.001). Group B also maintained significantly higher SpO₂ immediately after intubation (99.91 ± 0.28%) than group A (97.77 ± 2.24%; P < 0.001). No episodes of desaturation occurred in group B during the procedure (P < 0.001). In group A, 21.43% of patients desaturated to ≤ 95% (P < 0.001). Severe desaturation (SpO₂ < 92%) occurred in 7.14% of controls but was absent in group B (P = 0.023). Intubation time, intubation attempts, and bradycardia rates were comparable between groups (P > 0.05).

**Conclusions:**

Apneic oxygenation during intubation in children undergoing adenotonsillectomy effectively prevented desaturation without compromising safety or procedural efficiency

## 1. Background

Maintaining adequate oxygenation during the induction of general anesthesia is a major priority (1). Children are uniquely vulnerable to rapid oxygen desaturation because of their low functional residual capacity, high metabolic oxygen consumption, and increased alveolar ventilation relative to adults (2). These physiologic characteristics markedly shorten the safe apnea period and increase the risk of hypoxemia during airway manipulation (3). Even brief delays during laryngoscopy or intubation can result in precipitous declines in SpO₂, indicating the need for effective strategies that extend the duration of safe apnea (4).

Apneic oxygenation, defined as continuous oxygen delivery to the upper airway during periods without ventilation, has gained increasing attention as a technique to sustain oxygenation during induction (5). In adults, evidence has demonstrated that apneic oxygenation can prolong the time to desaturation, reduce hypoxemic events, and improve procedural safety (6). Its mechanism relies on the principle that oxygen continues to diffuse into the circulation despite absent ventilation, thereby maintaining alveolar oxygen stores and slowing the rate of hemoglobin desaturation (7).

However, evidence remains limited and variable, particularly in children (3). Adenotonsillar hypertrophy contributes to sleep-disordered breathing and chronic hypoventilation (8, 9). These factors further reduce baseline oxygen reserves and increase susceptibility to desaturation during apnea (2). Despite these risks, few studies have specifically evaluated apneic oxygenation in this high-risk population (3). Many pediatric trials exclude children with obstructive pathology, leaving a significant evidence gap in the population in which the potential benefit may be greatest (3, 10).

Clarifying the effectiveness of apneic oxygenation in children with compromised airway patency is essential for clinical practice (11). A simple, noninvasive intervention that reliably reduces desaturation events could meaningfully improve the safety of airway management during induction (3, 12-14). However, the degree of clinical benefit in partially obstructed pediatric airways remains uncertain, and existing data are insufficient to guide routine adoption.

## 2. Objectives

This prospective randomized controlled study aimed to evaluate the effectiveness of apneic oxygenation during the induction of general anesthesia in children undergoing adenotonsillectomy. We hypothesized that apneic oxygenation would decrease the frequency and severity of desaturation events and enhance overall oxygenation stability, providing a safer physiologic environment during airway instrumentation in this vulnerable population.

## 3. Methods

### 3.1. Study Design and Setting

This study was a prospective, single-blinded, randomized controlled superiority trial comparing apneic oxygenation with standard care during tracheal intubation in pediatric patients undergoing adenotonsillectomy. It was conducted at Fayoum University Hospital between December 2024 and June 2025 after approval from the local Institutional Ethics Committee and Institutional Review Board (protocol number: M 738). The trial was prospectively registered at ClinicalTrials.gov (Identifier: NCT06742476). Written informed consent was obtained from the parents or legal guardians of all eligible participants before recruitment and randomization. Participants were assigned in a 1:1 allocation ratio. No changes to the study protocol, eligibility criteria, interventions, or outcome measures were made after trial commencement.

### 3.2. Participants

Eligible participants were children aged 3 - 10 years of either sex who were classified as American Society of Anesthesiologists (ASA) physical status I or II and were scheduled to undergo adenotonsillectomy.

All enrolled children had adenotonsillar hypertrophy sufficient to warrant surgical removal and therefore exhibited mild to moderate upper-airway narrowing. Patients with severe obstruction, clinically significant obstructive sleep apnea, or anticipated difficult airway were excluded to avoid major confounders and maintain a homogeneous population. The study was designed to evaluate apneic oxygenation in the typical adenotonsillectomy population with partial, but not severe, airway obstruction.

Patients were excluded if parental consent was withheld, nasal intubation was required, or symptoms suggestive of nasal obstruction were present. Additional exclusion criteria included a history of respiratory disease, such as asthma or recent upper respiratory tract infection, congenital heart disease, or any anticipated difficulty with airway management. Children with syndromic features associated with difficult intubation, including Down syndrome, Goldenhar syndrome, or Pierre Robin sequence, were also excluded.

### 3.3. Randomization and Blinding

Participants were assigned to 1 of 2 groups. Group A (non-apneic oxygenation; n = 70) underwent tracheal intubation without apneic oxygenation, whereas group B (apneic oxygenation; n = 70) received continuous apneic oxygenation during tracheal intubation via a nasal cannula at a flow rate of 0.2 L/kg/min.

Randomization was performed using a computer-generated allocation sequence, and group assignments were placed in sequentially numbered, sealed, opaque envelopes that were opened only after patient enrollment and arrival in the operating room. Blinding was limited to the parents or legal guardians, who were not informed of group allocation. Blinding of the anesthesiologists performing intubation was not feasible because the intervention required visible placement of a nasal cannula during airway management.

### 3.4. Outcome Assessment

Oxygen saturation (SpO₂) and heart rate were monitored continuously using standard intraoperative monitoring, including pulse oximetry, and outcome values were recorded at predefined time points. The primary outcome was the lowest peripheral recorded during the intubation period, defined as the interval from cessation of bag-mask ventilation until successful endotracheal tube placement confirmed by the appearance of a square-wave capnographic trace. Oxygen saturation was monitored continuously by pulse oximetry throughout this period.

Secondary oxygenation outcomes included the number of patients whose SpO₂ decreased to predefined thresholds of ≤ 95%, ≤ 92%, and < 92% during the same interval. Threshold events were classified according to the lowest valid pulse oximeter reading recorded during intubation, with obvious signal artifact excluded on the basis of monitor signal quality and clinical correlation.

### 3.5. Procedure

Both groups received the same standardized general anesthesia protocol. After admission to the operating room, each child was positioned on the operating table, and standard monitoring was applied, including electrocardiography, noninvasive blood pressure measurement, pulse oximetry, and end-tidal carbon dioxide monitoring. Peri-induction adverse events, including bradycardia, laryngospasm, bronchospasm, aspiration, arrhythmia, and the need for rescue ventilation or additional airway maneuvers, were monitored and recorded.

Intravenous access was secured for fluid and medication administration. If intravenous access was initially difficult, anesthesia was induced with inhaled sevoflurane at 8% in oxygen with a fresh gas flow of 6 - 8 L/min, and intravenous cannulation was performed once an adequate depth of anesthesia had been achieved. Intravenous induction was then completed with propofol (2 - 3 mg/kg), fentanyl (1 mcg/kg), and atracurium (0.5 mg/kg). After induction, bag-mask ventilation with 100% oxygen at a flow of 6 - 8 L/min was provided for 3 minutes before ventilation was stopped.

In group B, a nasal cannula remained in place to deliver continuous apneic oxygenation at 0.2 L/kg/min throughout the apneic period during tracheal intubation. In group A, no supplemental oxygen was delivered during apnea. Intubation success was confirmed by bilateral chest auscultation and the presence of a square-wave capnographic trace, after which the nasal cannula was removed. Oxygen saturation was recorded immediately after intubation, and mechanical ventilation was initiated to maintain normocapnia and target oxygenation. If SpO₂ decreased to 92%, the intubation attempt was stopped, and bag-mask ventilation with 100% oxygen was resumed until SpO₂ returned to 100%. Intubation was then completed by the supervising consultant anesthesiologist.

### 3.6. Outcome Measures

The primary outcome was the lowest SpO₂ recorded during tracheal intubation. Secondary outcomes included the number of patients whose SpO₂ decreased to 95%, 92%, or below 92%. Additional secondary measures were baseline SpO₂, SpO₂ after bag-mask ventilation, and SpO₂ immediately following intubation. The total number of intubation attempts and the duration of intubation, measured from cessation of bag-mask ventilation to successful tube placement, were also recorded. The incidence of bradycardia, defined as a decrease in heart rate of more than 20% from baseline, was documented.

### 3.7. Sample Size Calculation

The required sample size was calculated before the start of the study using G*Power version 3.1.9.6. The calculation was informed by findings from Ray et al. (13, 15), who reported a 1.58% difference in the lowest SpO₂ between control and apneic oxygenation groups, with corresponding standard deviations of 4.6% and 0.29%. These values were used to derive an estimated effect size of 0.48 for the primary outcome. Based on this effect size, a 2-tailed alpha level of 0.05, statistical power of 80%, and equal allocation between the 2 groups, the software indicated that a minimum of 68 patients was needed per arm. To maintain adequate power and account for potential dropouts, the target enrollment was increased to 70 children in each group, resulting in a total sample of 140 participants.

### 3.8. Statistical Analysis

Statistical analysis was performed using SPSS version 23 (IBM Corp, Armonk, NY, USA). Continuous variables were assessed for normality using the Shapiro-Wilk test before inferential analysis and are presented as mean ± standard deviation. Between-group comparisons of normally distributed continuous variables were performed using the independent-samples Student *t*-test. Categorical variables are presented as number and percentage and were compared using the Chi-square test. The between-group difference in the primary outcome, lowest SpO₂ during intubation, was additionally expressed as the mean difference with its 95% confidence interval. No formal adjustment for multiple comparisons was applied to secondary outcomes; therefore, these analyses should be interpreted cautiously. All tests were 2-sided, and a P-value < 0.05 was considered statistically significant.

## 4. Results

**Figure 1.**
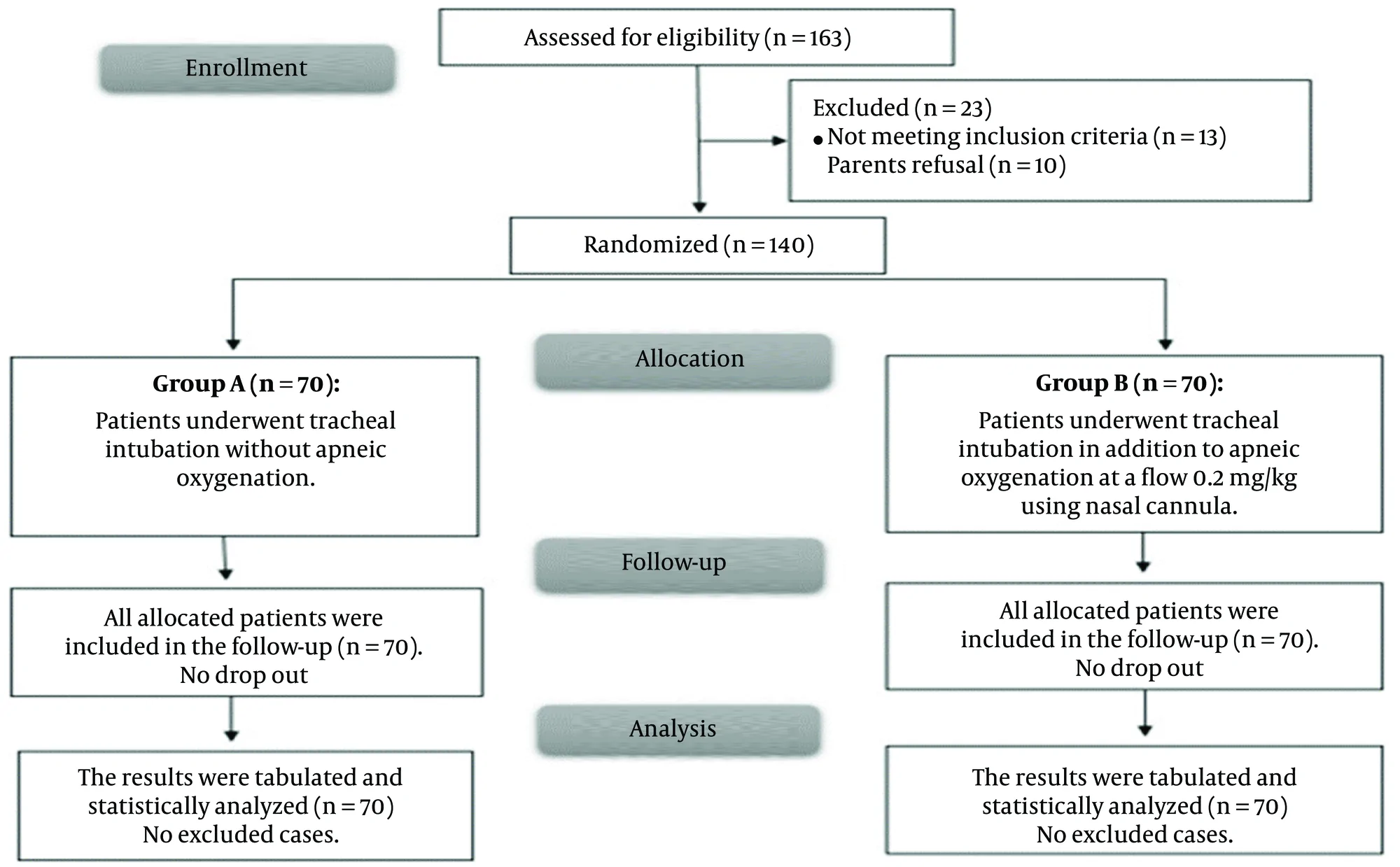
CONSORT flow diagram illustrating patient screening, assessment for eligibility, randomization, group allocation, and inclusion in the final analysis.

A total of 163 children were assessed for eligibility between December 2024 and June 2025. Thirteen patients did not meet the inclusion criteria, and 10 declined participation. The remaining 140 patients were randomized, with 70 allocated to group A and 70 allocated to group B. All randomized patients received the allocated intervention as planned and were included in the analysis of the primary and secondary outcomes. No patients were excluded after randomization. The participant flow is summarized in Figure 1. 

### 4.1. Baseline Characteristics

Demographic variables and clinical characteristics, including age, sex, weight, height, Body Mass Index (BMI), and ASA physical status, were similar between groups, with no statistically significant differences observed (Table 1). 

**Table 1.** Demographic and Clinical Characteristics of the Studied Groups ^a^

Variables	Non-apneic Oxygenation Group A (N = 70)	Apneic Oxygenation Group B (N = 70)	P-Value
**Age (y)**			0.305
Mean ± SD	5.76 ± 1.85	6.06 ± 1.59	
Range	3 - 10	4 - 9	
**Sex**			0.604
Male	44 (62.86)	41 (58.57)	
Female	26 (37.14)	29 (41.43)	
**Weight (kg)**			0.157
Mean ± SD	24.53 ± 5.47	25.79 ± 4.96	
Range	16 - 37	19 - 34	
**Height (cm)**			0.149
Mean ± SD	114.21 ± 11.69	116.97 ± 10.79	
Range	96 - 141	102 - 135	
**BMI (kg/m²)**			0.061
Mean ± SD	21.6 ± 3.37	22.7 ± 3.48	
Range	16 - 27	17 - 28.8	
**ASA Physical Status**			0.127
I	18.55 ± 0.56	18.66 ± 0.28	
II	16.8 - 20	17.4 - 18.9	

Abbreviations: ASA, American Society of Anesthesiologists; BMI, body mass index; SD, standard deviation.

^a^ Data are presented as mean ± SD or number (%).

### 4.2. Primary Outcome

**Figure 2.**
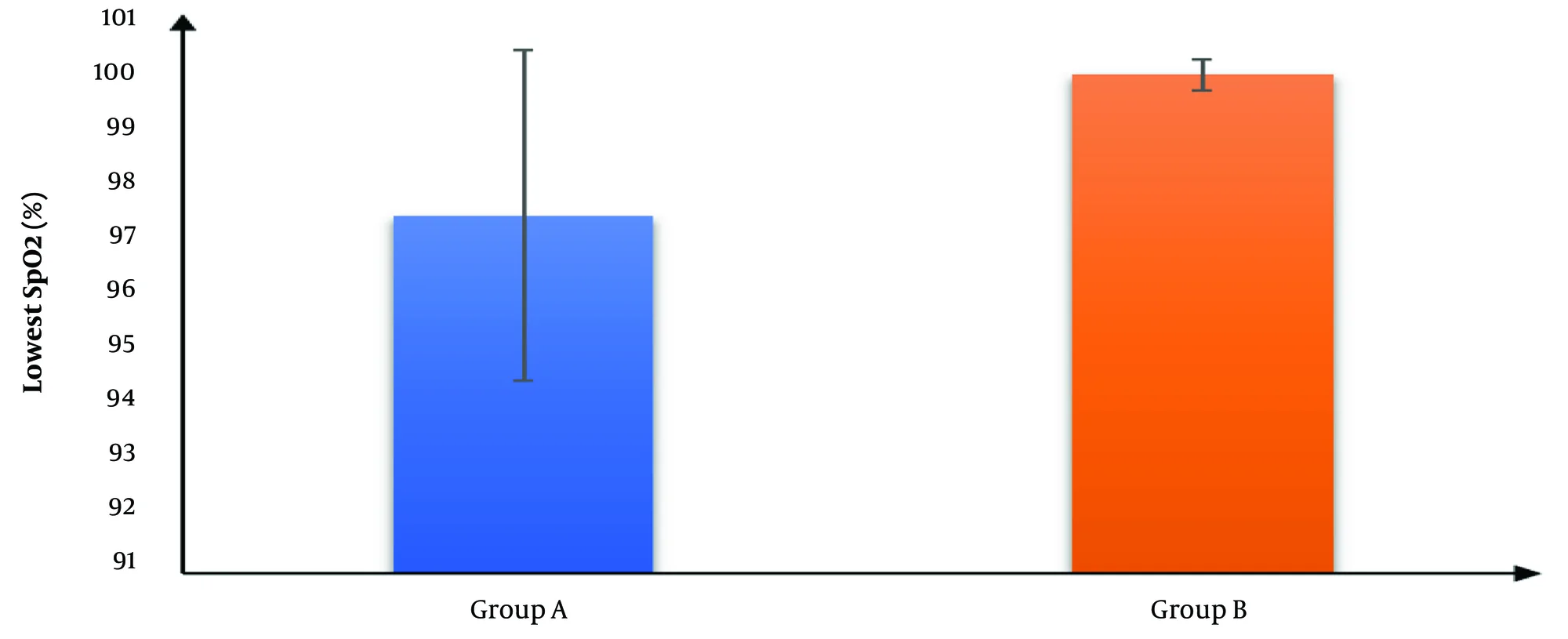
Comparison of the lowest peripheral oxygen saturation (SpO₂) recorded during tracheal intubation between the non-apneic oxygenation group (group A) and the apneic oxygenation group (group B).

The lowest SpO₂ during tracheal intubation was significantly lower in the non-apneic oxygenation group (group A) than in the apneic oxygenation group (group B). Group A recorded a mean SpO₂ of 97.40 ± 2.96%, while group B maintained a mean SpO₂ of 99.91 ± 0.28% (P < 0.001) (Table 2 and Figure 2). 

**Table 2.** Lowest Peripheral Oxygen Saturation in the Studied Groups

Variables	Non-apneic Oxygenation Group A (N = 70)	Apneic Oxygenation Group B (N = 70)	P-Value
**Lowest SpO₂ (%)**			< 0.001
Mean ± SD	97.40 ± 2.96	99.91 ± 0.28	
Range	90 - 100	99 - 100	

Abbreviations: SD, standard deviation; SpO₂, peripheral oxygen saturation.

### 4.3. Secondary Oxygenation Outcomes

Immediately after intubation, SpO₂ was again significantly lower in group A (97.77 ± 2.24%) than in group B (99.91 ± 0.28%) (P < 0.001) (Table 3). 

**Table 3.** Peripheral Oxygen Saturation Immediately After Intubation ^a^

Spo₂ (%)	Non-Apneic Oxygenation Group A (N = 70)	Apneic Oxygenation Group B (N = 70)	P-Value
**Baseline**	99.73 ± 0.45	99.57 ± 0.50	0.052
**After bag-mask ventilation**	99.90 ± 0.3	99.91 ± 0.28	0.773
**Immediately after intubation**	97.77 ± 2.24	99.91 ± 0.28	< 0.001

Abbreviations: SD, standard deviation; SpO₂, peripheral oxygen saturation.

^a^ Data are presented as mean ± SD.

**Figure 3.**
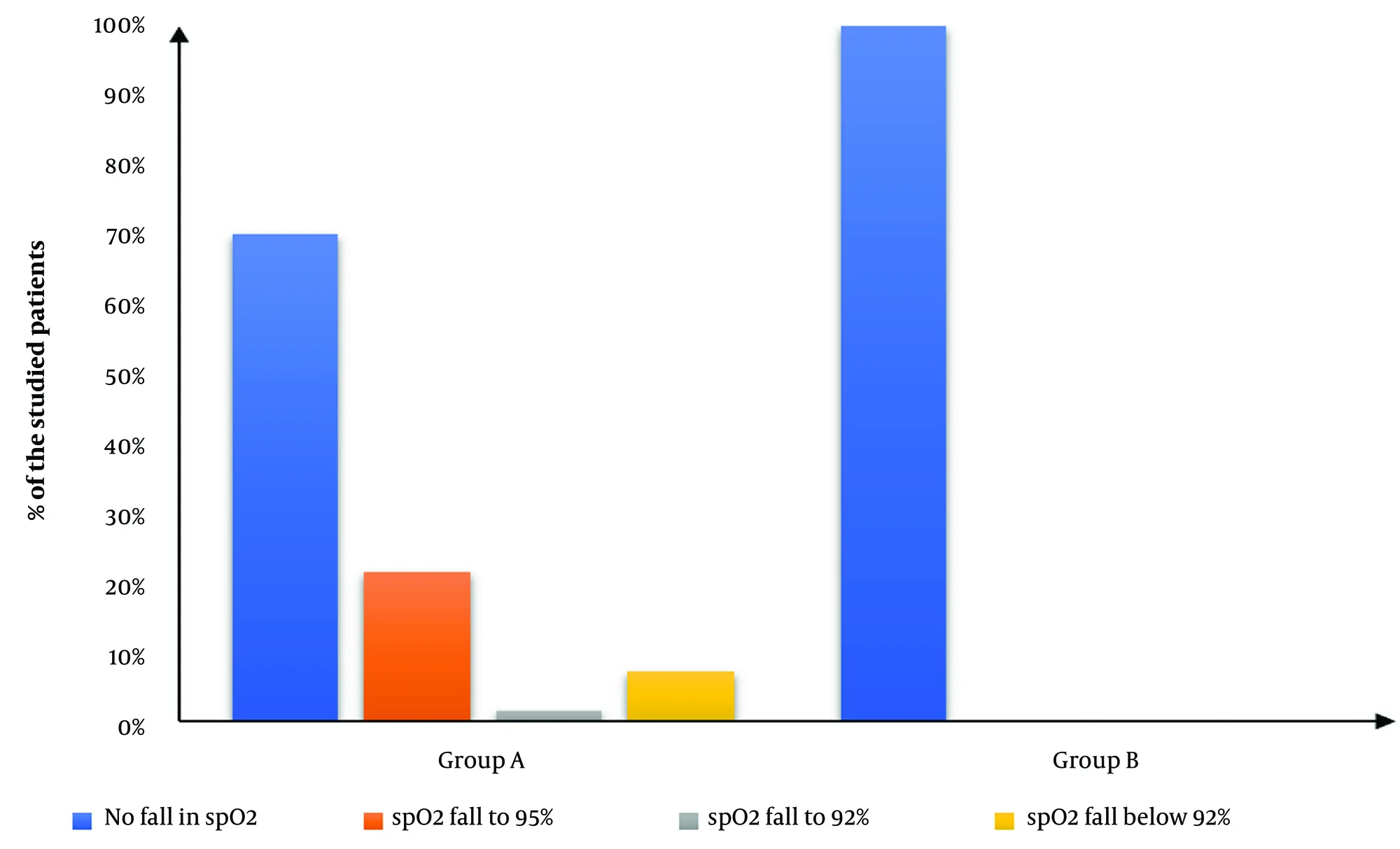
Distribution of desaturation events during laryngoscopy and tracheal intubation in the 2 study groups, shown as the number of patients with peripheral oxygen saturation (SpO₂) ≤ 95%, ≤ 92%, and < 92%.

During laryngoscopy and intubation, no episodes of desaturation occurred in group B, whereas 21 children in group A experienced a decrease in SpO₂ (P < 0.001). Of these, 15 children desaturated to 95%, 1 child desaturated to 92%, and 5 children desaturated to below 92% (Table 4 and Figure 3). 

**Table 4.** Decrease in Peripheral Oxygen Saturation During Laryngoscopy and Intubation ^a^

SpO₂ Category	Non-apneic Oxygenation Group A (N = 70)	Apneic Oxygenation Group B (N = 70)	P-Value
**No fall in SpO₂**	49 (70)	70 (100)	< 0.001
**SpO₂ fall to 95%**	15 (21.43)	0 (0)	< 0.001
**SpO₂ fall to 92%**	1 (1.43)	0 (0)	0.316
**SpO₂ fall below 92%**	5 (7.14)	0 (0)	0.023

Abbreviation: SpO₂, peripheral oxygen saturation.

^a^ Data are presented as number (%).

### 4.4. Intubation Time and Attempts

Mean intubation time was slightly longer in group B (66.97 ± 18.88 seconds) than in group A (63.04 ± 22.73 seconds), but the difference was not statistically significant (P = 0.268). The number of intubation attempts also did not differ significantly between groups (Table 5). Five children in group A who desaturated below 92% required intervention by the supervising consultant anesthesiologist and were subsequently intubated on the second attempt.

**Table 5.** Intubation Time and Number of Intubation Attempts in the Studied Groups ^a^

Variables	Non-apneic Oxygenation Group A (N = 70)	Apneic Oxygenation Group B (N = 70)	P-Value
**Intubation time (s)**			0.268
Mean ± SD	63.04 ± 22.73	66.97 ± 18.88	
Range	22 - 100	32 - 100	
**Number of intubation attempts**			0.085
One attempt	57 (81%)	64 (91%)	
Two attempts	13 (19%)	6 (9%)	
**Bradycardia**			0.718
Yes	5 (7.14%)	3 (4.29%)	
No	65 (92.86%)	67 (95.71%)	

Abbreviation: SD, standard deviation.

^a^ Data are presented as number (%) unless otherwise indicated.

### 4.5. Bradycardia

With respect to safety, bradycardia was the only adverse event observed and did not differ significantly between the 2 groups. No episodes of laryngospasm, bronchospasm, aspiration, clinically significant arrhythmia, or other airway-related complications were recorded in either group. When SpO₂ decreased to 92%, intubation was interrupted, and bag-mask ventilation with 100% oxygen was resumed according to the study protocol until SpO₂ returned to 100%.

## 5. Discussion

Apneic oxygenation, first described in the 1950s as a method to maintain oxygenation during apnea, has recently regained interest with the use of high-flow oxygen systems (16). Despite its strong physiological rationale, its application in pediatric patients remains relatively understudied (17).

In this single-blinded randomized controlled trial, apneic oxygenation effectively improved oxygenation during tracheal intubation in children undergoing adenotonsillectomy without affecting procedural safety. Children receiving apneic oxygenation maintained more stable SpO₂ throughout airway instrumentation, while intubation time, number of attempts, and bradycardia incidence remained comparable between groups.

Although the difference in mean SpO₂ values between groups was relatively small, this should be interpreted within the physiological context of pediatric SpO₂, where values are typically close to the upper limit. Consequently, small numerical differences may mask clinically relevant events. Our findings showed that desaturation episodes occurred only in the control group, whereas no child receiving apneic oxygenation experienced clinically significant oxygen decline. This suggests that apneic oxygenation provides an important safety margin during apnea in children with partially obstructed airways.

To our knowledge, this study is among the first randomized trials evaluating apneic oxygenation specifically in children with adenotonsillar hypertrophy, a population known to be particularly vulnerable to peri-intubation desaturation.

Sachin et al. (14, 18), in a trial of 116 children, reported higher SpO₂ levels and fewer hypoxic events in the apneic oxygenation group, consistent with the improved oxygen stability observed in our cohort. Similar results were noted by Olayan et al. (11, 19), who found that all children receiving apneic oxygenation at 3 L/min maintained 100% saturation, while 1 child in the standard care group experienced profound desaturation to 73%. Shetty et al. (15, 20) also showed that infants receiving apneic oxygenation sustained 100% SpO₂ throughout intubation.

Our results also align with higher-quality evidence. Fuchs et al. (5) demonstrated in a meta-analysis of 15 studies that apneic oxygenation significantly reduces severe hypoxemia, reporting a 58% reduction in SpO₂ < 90% events. Ray et al. (13, 15) further confirmed the protective effect in a prospective randomized controlled trial of 106 children, showing that nasal cannula-assisted apneic oxygenation effectively prevents desaturation during laryngoscopy and intubation. Evidence from Aroonpruksakul et al. (1, 21) extends this benefit to rapid sequence induction settings, emphasizing reduced peri-intubation desaturation in high-risk scenarios.

In our study, no child in the apneic oxygenation group experienced a decrease in SpO₂ below 95%, whereas 21 children in the control group desaturated, reinforcing the robust protection described by Fuchs et al. (5). Importantly, apneic oxygenation did not prolong intubation time or increase the number of attempts, consistent with Olayan et al. (11, 19), who similarly found no procedural delay despite improved oxygenation outcomes. In contrast, Soneru et al. (17, 22) observed a significant difference in intubation attempts between study groups when intubation was performed by inexperienced trainees, which differs from our finding of an insignificant difference in the number of intubation attempts. This may be explained by the efficiency and competence of the anesthesiologists in our study, although Soneru et al. (22) observed fewer attempts among inexperienced trainees in their setting. The low and comparable incidence of bradycardia between groups in our trial further supports the safety of this technique.

Anesthesiologists must adapt oxygenation and intubation techniques to maintain stability throughout induction in children (23-25). Apneic oxygenation maintains oxygen diffusion into the bloodstream despite the absence of active ventilation, thereby prolonging the safe apnea period and reducing the risk of hypoxemia during airway instrumentation (3, 25, 26).

This randomized controlled trial used a standardized anesthesia protocol with complete follow-up for all enrolled patients, providing reliable peri-intubation data on apneic oxygenation in children undergoing adenotonsillectomy. However, several limitations should be considered. The study was conducted at a single center, which may limit generalizability. Arterial blood gas analysis was not performed because of the minor nature and cost of the procedure, preventing direct evaluation of PaO₂ and PaCO₂ during apnea. Additionally, objective measures of airway obstruction, such as tonsillar grade or formal airway difficulty scores, were not recorded, which may limit comparison across children and restrict generalizability. Intubating clinicians were not blinded to group allocation, introducing potential performance bias. Capnography was not monitored during apnea, limiting assessment of carbon dioxide accumulation, and outcomes were restricted to short-term peri-intubation parameters without postoperative respiratory follow-up.

Despite these limitations, our findings indicate that low-flow apneic oxygenation at 0.2 L/kg/min via nasal cannula effectively prevents desaturation in children undergoing adenotonsillectomy with partial upper-airway obstruction without prolonging intubation or increasing adverse events, supporting its routine use in this setting. Apneic oxygenation is already known to reduce hypoxemia during pediatric intubation, although existing evidence largely comes from heterogeneous populations and often excludes children with adenotonsillar hypertrophy or other obstructive pathology. Therefore, the findings of this study are most applicable to children undergoing adenotonsillectomy with partial upper-airway obstruction and may not be generalizable to those with severe obstruction, clinically significant obstructive sleep apnea, obesity, syndromic airway abnormalities, or anticipated difficult airway. Future studies should evaluate its use in higher-risk subgroups, such as obese children and those with mild obstructive sleep apnea, assess longer apnea durations during difficult intubations, include broader age ranges, and incorporate arterial blood gas analysis to better characterize PaO₂ and PaCO₂ changes.

### 5.1. Conclusions

This study indicates that apneic oxygenation is a simple, low-risk intervention that meaningfully reduces hypoxemia during pediatric intubation. Widespread adoption could improve patient safety and outcomes in routine and challenging airway scenarios.

## Data Availability

The datasets generated and analyzed during this study are not publicly available, but they may be obtained from the corresponding author upon reasonable request.

## Clinical Trial Registration Code:

The trial was prospectively registered at ClinicalTrials.gov (Identifier: NCT06742476).
